# Free Testosterone Reflects Metabolic as well as Ovarian Disturbances in Subfertile Oligomenorrheic Women

**DOI:** 10.1155/2018/7956951

**Published:** 2018-09-10

**Authors:** L. Antonio, S. Pauwels, M. R. Laurent, D. Vanschoubroeck, I. Jans, J. Billen, F. Claessens, B. Decallonne, Diane De Neubourg, P. Vermeersch, D. Vanderschueren

**Affiliations:** ^1^Department of Chronic Diseases, Metabolism and Ageing (CHROMETA), Laboratory of Clinical and Experimental Endocrinology, KU Leuven, Leuven, Belgium; ^2^Department of Endocrinology, University Hospitals Leuven, Leuven, Belgium; ^3^Department of Laboratory Medicine, University Hospitals Leuven, Leuven, Belgium; ^4^Department of Cardiovascular Sciences, KU Leuven, Leuven, Belgium; ^5^Department of Cellular and Molecular Medicine, Laboratory of Molecular Endocrinology, KU Leuven, Leuven, Belgium; ^6^Department of Obstetrics and Gynecology, University Hospitals Leuven, Leuven, Belgium; ^7^Department of Reproductive Medicine, Antwerp University Hospital, Edegem, Belgium

## Abstract

**Background:**

Diagnosing polycystic ovary syndrome (PCOS) is based on ovulatory dysfunction, ovarian ultrasound data, and androgen excess. Total testosterone is frequently used to identify androgen excess, but testosterone is mainly bound to sex hormone-binding globulin (SHBG) and albumin. Only 1-2% of nonprotein-bound testosterone (so-called free testosterone) is biologically active and responsible for androgen action. Moreover, automated immunoassays which are frequently used for female testosterone measurements are inaccurate.

**Objective:**

To assess the clinical usefulness of liquid chromatography-tandem mass spectrometry measured testosterone and calculated free testosterone in subfertile women attending a fertility clinic with oligomenorrhea and suspected PCOS.

**Methods:**

Hormonal and metabolic parameters were evaluated, and ovarian ultrasound was performed. Total testosterone was measured by liquid chromatography-tandem mass spectrometry. Free testosterone was calculated from total testosterone and SHBG.

**Results:**

Sixty-six women were included in the study. Total testosterone was associated with ovarian volume and antral follicle count but not with metabolic parameters. However, SHBG and calculated free testosterone were associated with both ovarian ultrasound and metabolic parameters, such as BMI and insulin resistance.

**Conclusions:**

Assessing SHBG and free testosterone is important in evaluating androgen excess in subfertile women with ovulatory dysfunction and suspected PCOS, as it reflects both ovarian and metabolic disturbances.

## 1. Introduction

Polycystic ovary syndrome (PCOS) is very common in women with subfertility and oligomenorrhea [1–3]. Diagnosing PCOS is based on the “Rotterdam criteria”: the presence of at least two of the following conditions: ovulatory dysfunction, polycystic ovary morphology (PCOM) on ultrasound, or androgen excess [4]. Although very frequently used, these criteria have important limitations. Due to improved ultrasound imaging, PCOM is often present, also in normally cycling women without other PCOS features. Furthermore, these diagnostic criteria do not take into account metabolic parameters, and patients with clearly different metabolic characteristics are diagnosed under the umbrella term of PCOS [5, 6].

Diagnosing androgen excess in women can also be challenging, as it may be assessed either clinically (hirsutism or acne), biochemically, or both [2, 4]. It remains unclear which androgen(s) should be measured to assess biochemical hyperandrogenemia in women suspected for PCOS [7]. In clinical practice, total testosterone (total T) is frequently used, but measuring total T levels in women by automated immunoassays (IA) is often inaccurate. Instead of IA, liquid chromatography-tandem mass spectrometry (LC-MS/MS) has therefore been proposed as the method of choice for accurate measurement of low testosterone levels in women [8–11]. Also, androstenedione (A4), the steroid precursor of testosterone, has been proposed as a marker of androgen excess in PCOS patients, especially in identifying PCOS patients with a higher metabolic risk [12].

Furthermore, testosterone is mainly bound to sex hormone-binding globulin (SHBG) and albumin. Only a small fraction (1-2%) circulates as nonprotein-bound free testosterone (free T), and it is only the free T fraction that can enter the cell and exert androgen activity [13].

In a recent best practice summary, free T was proposed as the most sensitive marker for diagnosing androgen excess [10] and equilibrium dialysis as the preferred measurement method. However, this technique is only available in a limited number of reference laboratories [10, 14]. Instead of a direct free T measurement, calculated free T can be used to determine hyperandrogenemia in PCOS patients [9, 10]. Inevitably, these calculations require an accurate measurement of total T and SHBG [2]. Fortunately, when LC-MS/MS-measured total T is used, there is an excellent correlation between calculated free T and measured free T in women, and calculated free T can be used to evaluate female androgen status [9, 10, 15].

However, to date, only a limited number of studies have investigated the clinical correlates of these newly emerging LC-MS/MS measurements in the appraisal of female androgen status. Specifically, the use of LC-MS/MS-measured total T, A4, and free T (calculated from LC-MS/MS total T) is not well established in the diagnosis of PCOS. Nevertheless, accurately diagnosing hyperandrogenism is important, as women with androgen excess are at increased risk of developing type 2 diabetes and the metabolic syndrome [16].

In this study, we assessed the clinical usefulness of using state-of-the-art LC-MS/MS technology to measure sex steroids in subfertile women with oligomenorrhea and suspected PCOS. Furthermore, associations between total and free testosterone and metabolic and ovarian parameters were analyzed.

## 2. Methods

### 2.1. Subjects

97 women with oligo- or amenorrhea (cycle length > 38 days) were recruited at the Leuven fertility center. All women were screened for pregnancy and congenital adrenal hyperplasia (21-hydroxylase deficiency). Women taking oral contraceptives were excluded. Subjects with hyperprolactinemia (*n* = 1), newly diagnosed type 2 diabetes (*n* = 1), active thyroid disease (*n* = 1), hypothalamic amenorrhea (luteinizing hormone (LH), follicle-stimulating hormone (FSH) below the lower limits of the reference intervals (<2.4 U/L for LH and <3.5 U/L for FSH), *n* = 1), or premature ovarian failure (FSH > 12 U/L) (*n* = 2) were excluded. Furthermore, 3 women were excluded because of an ovulatory (LH > 40 U/L) or luteal blood sample (progesterone > 1.5 *μ*g/L). In 22 women, there was no serum available for additional sex steroid measurements, and these women were also excluded, leaving 66 women in the study sample (Supplementary Figure 1).

The study protocol was approved by the local ethical board of the University Hospitals Leuven. All patients gave written informed consent.

### 2.2. Clinical Assessments

At inclusion, weight, height, waist circumference, and blood pressure were recorded for all patients. BMI was calculated from weight and height. Hirsutism was assessed with the simplified Ferriman-Gallwey score, and patients were classified with hirsutism if this score was ≥3 [17]. Acne was self-reported. Patients with a BMI < 25 kg/m^2^ were classified as having a normal BMI; patients with a BMI between 25 and 29.9 were classified as overweight, and those with a BMI ≥ 30 as obese.

### 2.3. Sex Steroid Measurements by Liquid Chromatography-Tandem Mass Spectrometry

Estradiol (E2) and estrone (E1) were measured by LC-MS/MS as described previously [18]. Total T and A4 were measured by a newly developed LC-MS/MS method. Method details are described in Supplementary Materials. Free testosterone was calculated with the Vermeulen formula [19]. LC-MS/MS measurements were compared with originally reported values by direct immunoassay (Diasorin Gamma Coat) for A4 and electrochemiluminescence immunoassay (ECLIA) on a Modular E platform (Roche Diagnostics) for total T for all patients if available in the medical records (*n* = 57).

### 2.4. Other Laboratory Measurements

LH, FSH, progesterone, SHBG, thyroid-stimulating hormone (TSH), dehydroepiandrosterone sulphate (DHEAS), and fasting insulin were measured by ECLIA (Modular E170 from Roche Diagnostics). Anti-Mullerian hormone (AMH) was measured by enzyme-linked immunosorbent assay (ELISA) (Beckman Coulter Gen II). Fasting glucose, total cholesterol, HDL cholesterol, and triglycerides were measured by a colorimetric method (Cobas c702 from Roche Diagnostics). LDL cholesterol was calculated from total cholesterol, HDL cholesterol, and triglycerides by the Friedewald formula [20]. The LH/FSH ratio was calculated by dividing the LH concentration in U/L by the FSH concentration in U/L. Insulin resistance was calculated using the updated homeostasis model assessment of insulin resistance (HOMA-IR) [21].

### 2.5. Ovarian Ultrasound

Two-dimensional vaginal ultrasound was performed by an experienced gynaecologist (Voluson E8, GE Healthcare). For both ovaries, the number of antral follicles (AFC) was counted, from which the mean AFC was calculated [22]. For both ovaries, ovarian volume (OV) was calculated (0.5 × length × width × thickness). The mean volume of the left and right ovary was also calculated. Furthermore, follicle localization (random, peripheral, or both) and follicle size (uniform or nonuniform) were registered.

Thirteen patients with a dominant follicle, a corpus luteum, a hemorrhagic cyst, or a history of ovarian surgery or teratoma were additionally excluded when assessing ultrasound parameters. Patients were classified as having PCOM if they had ≥12 antral follicles (2–9 mm in diameter) in both ovaries and/or an ovarian volume > 10 mL in one or two ovaries [4, 23].

### 2.6. Polycystic Ovary Syndrome (PCOS) Definition

Polycystic ovary syndrome was defined by the Rotterdam criteria: the presence of at least two of the following criteria: ovulatory dysfunction, PCOM, or biochemical androgen excess [4]. PCOM was defined as discussed above. For androgen excess, total T > 41 ng/dL or free T > 0.49 ng/dL was used as cutoff [15].

### 2.7. Statistical Analysis

Spearman rank was used to assess correlations between hormonal measurements. Linear or logistic regression (unadjusted and adjusted for age and BMI) was used to assess associations between androgens, SHBG, and metabolic and ultrasound parameters. Pearson's *r* was used to assess correlations between LC-MS/MS and immunoassay results and ultrasound parameters. *P* < 0.05 was considered statistically significant. All analyses were performed using the STATA version 13 (Stata Corp).

## 3. Results

Age, hormonal, metabolic, and ovarian parameters of patients are reported in Table 1. Correlations between the different hormonal measurements are shown in Table 2. As expected, total T, A4, and free T were strongly correlated with each other. Furthermore, E2 was correlated with total T and A4, whereas E1 was also correlated with free T. LH and LH/FSH ratio were related to total and free T and A4. Furthermore, LC-MS/MS androgen measurements showed a better correlation with ultrasound data than immunoassay measurements (AFC and ovarian volume; *r* = 0.49 and 0.51 for LC-MS/MS total T; *r* = 0.42 and 0.41 for immunoassay total T; *r* = 0.55 and 0.58 for LC-MS/MS A4; *r* = 0.44 and 0.56 for radioimmunoassay A4, data not shown).

Associations between androgens, SHBG, and metabolic and ultrasound parameters are shown in Table 3. Total T, A4, and free T closely reflected ovarian volume and AFC in oligomenorrheic subfertile patients, independent of BMI. Neither total T nor A4 was related to BMI, insulin, or insulin resistance. In contrast, increasing free T or decreasing SHBG concentrations were associated with a higher BMI, as well as higher insulin levels and insulin resistance, but this association disappeared after adjusting for BMI. After adjusting for age and BMI, total testosterone was associated with total cholesterol and LDL cholesterol. There were no significant associations between free T and lipid measurements after adjustments for age and BMI.

Thirty-seven women (56%) had normal free T (≤0.49 ng/dL), and 29 women (44%) had high free T (>0.49 ng/dL) (Table 4). Total T, A4, LH, and LH/FSH levels were higher in women with high free T, whereas SHBG was lower. All the observed differences remained significant after adjusting for age and BMI (Table 4). Women with high free T had a higher BMI, had higher insulin levels, and were more insulin-resistant. However, the associations between free T and insulin or HOMA-IR disappeared after adjusting for age and BMI. Furthermore, patients with high free T had a higher AMH level, a higher mean ovarian volume, and an increased number of antral follicles, also after adjusting for age and BMI (Table 4). In Supplementary Table 1, the same comparisons were made between women with normal total T (≤41 ng/dL) and high total T (>41 ng/dL).

## 4. Discussion

In our study, total T, A4, and free T closely reflected ovarian volume and AFC in oligomenorrheic subfertile patients with suspected PCOS. However, neither total T nor A4 was related to BMI, insulin, or insulin resistance. In contrast, increasing free T concentrations was associated with a higher BMI, as well as higher insulin levels and insulin resistance [24], but this association disappeared after adjusting for BMI.

This link between free T and metabolic parameters can, at least partly, be explained by the impact of BMI on SHBG levels. As expected, SHBG was inversely associated with BMI, and it is well known that SHBG levels decrease in obesity, both in men and women. In women, an obesity-related decrease in SHBG is accompanied by a higher free T. This is in contrast to obese men, in whom a decrease in SHBG is accompanied by a decrease in total T, whereas free T remains normal or slightly decreases [25, 26]. Furthermore, SHBG levels in women are two to three times higher than in men, as is the number of unoccupied SHBG steroid-binding sites (up to 80% in women versus 45% in men) [27, 28]. Hence, the sex steroid buffering capacity of SHBG is higher in women than in men, and the main function of SHBG in women is protection against high free androgen levels [29]. Thus, when SHBG levels decrease with increasing BMI, this buffering capacity is breached, eventually leading to increasing free T concentrations and androgen excess. It is therefore likely that even slight changes in the biological availability of androgens may have clinical consequences in women.

Furthermore, free T was positively correlated with LH and LH/FSH ratio, and women with high free T have higher LH and a higher LH/FSH ratio compared to women with normal free T, independent of BMI. Androgen excess can dysregulate hypothalamic-pituitary-ovarian axis function by disrupting normal GnRH pulse frequency. As a result, pituitary LH production increases, while FSH remains inadequately low, eventually hampering cyclic ovarian estradiol and progesterone production [30]. Oligomenorrhea and ovulatory dysfunction may thus be early clinical signs of androgen excess. Moreover, women with high free T levels had a higher AMH, higher ovarian volume, and higher AFC, independent of BMI. Thus, women with high free T have numerous characteristics of PCOS: ovarian aspects (an increase in AMH as well as ultrasound features), hormonal aspects (a higher LH/FSH ratio), and also metabolic aspects (BMI, insulin levels, and insulin resistance). A similar analysis for women with high total T showed nonsignificant unadjusted *P* values for metabolic parameters (BMI, insulin levels, and insulin resistance; *P* = 0.382, 0.639, and 0.632, resp.).

Our study has several strengths. All sex steroids were measured by a sensitive LC-MS/MS method, suitable for precise measurement of low testosterone concentrations in women. In concordance with literature [11, 15], direct immunoassay results misclassified almost 1 out of 4 patients as compared to LC-MS/MS measurements, reflecting the nonideal correlation for total T as well as A4 (Pearson's *r* = 0.77 and 0.68, resp.). This further supports the use of validated LC-MS/MS methods in evaluating women for biochemical hyperandrogenemia [15, 31]. In addition, extensive sample pretreatment is not needed and only 200 *μ*L of serum is required; therefore, our LC-MS/MS method is applicable for measuring total T and A4 in routine clinical practice. Furthermore, in all patients, ovarian ultrasound was rigorously performed, and observations were recorded in a standardized manner. Additionally, a broad range of hormonal and metabolic parameters was registered.

However, some limitations need to be considered. Our study sample is relatively small, and only oligomenorrheic patients consulting a university fertility center were included. Although 45% of patients are overweight or obese, most patients have a favorable metabolic profile. Our findings therefore need validation in other patient groups. Due to the cross-sectional and observational design of the study, we cannot discriminate between cause and effect.

In conclusion, assessing SHBG and free testosterone is important in evaluating androgen excess in subfertile women with ovulatory dysfunction and suspected PCOS, as it reflects both ovarian and metabolic disturbances.

## Figures and Tables

**Table 1:** Biochemistry, hyperandrogenism, metabolic and ovarian parameters, and PCOS diagnosis for 66 women with oligomenorrhea.

	Mean (SD) or *n* (%)	Reference interval	Limit of quantification
Age	28.3 (3.0)		
Biochemistry			
Total T (ng/dL)	46.5 (23.7)	≤41	2.5
A4 (ng/dL)	180 (90)	≤240	2.5
Free T (ng/dL)	0.57 (0.37)	≤0.49	
SHBG (nmol/L)	70.1 (32.6)	41–103	2.0
E2 (ng/L)	59.2 (50.6)	15–350	1.3
E1 (ng/L)	61.9 (33.4)	17–200	1.2
LH (U/L)	9.4 (4.8)	2.4–12.6	0.1
FSH (U/L)	5.8 (1.3)	3.5–12.5	0.1
LH/FSH	1.6 (0.9)		
DHEAS (*μ*g/dL)	212 (91)	98.8–340	0.1
AMH (ng/mL)	8.4 (5.4)	1.0–9.5	0.03
Clinical hyperandrogenism			
Hirsutism score	2.1 (2.4)	<3	
Having hirsutism	21 (33%)		
Having acne	28 (44%)		
Metabolic parameters			
BMI	25.0 (5.2)	18.5–24.9	
% normal BMI	36 (54.6%)		
% overweight	21 (31.8%)		
% obese	9 (13.6%)		
Waist circumference (cm)	86.4 (13.3)	<80	
Glucose (mg/dL)	92.2 (15.7)	80–110	2
Insulin (pmol/L)	67.4 (40.6)	17.8–173	0.3
HOMA-IR	1.25 (0.74)		
Total cholesterol (mg/dL)	174.6 (29.6)	≤190	3.9
HDL cholesterol (mg/dL)	52.0 (14.2)	≥45	3.1
LDL cholesterol (mg/dL)	104.6 (25.0)	≤115	
Triglycerides (mg/dL)	90.1 (50.2)	≤150	8.8
Ovarian ultrasound parameters (*n* = 53)			
Mean ovarian volume (mL)	10.0 (4.1)	≤10	
Mean number of antral follicles	31.0 (14.8)	<12	
Follicle localization			
Random	29 (59%)		
Peripheral	18 (37%)		
Random and peripheral	2 (4%)		
Follicle size			
Uniform	16 (37%)		
Nonuniform	27 (63%)		
% of women meeting PCOM criteria	49 (92%)		
PCOS diagnosis (*n* = 53)			
Having PCOS	49 (92%)		
Oligomenorrhea + PCOM + high total T	24 (49%)		
Oligomenorrhea + PCOM + normal total T	25 (51%)		

For ultrasound parameters and PCOS definition: 13 patients were additionally excluded (see Methods). Total T: total testosterone; A4: androstenedione; E2: estradiol; E1: estrone; free T: calculated free testosterone; SHBG: sex hormone-binding globulin; LH: luteinizing hormone; FSH: follicle-stimulating hormone; DHEAS: dehydroepiandrosterone sulphate; AMH: anti-Mullerian hormone; BMI: body mass index; HOMA-IR: homeostasis model assessment of insulin resistance; PCOM: polycystic ovary morphology.

**Table 2:** Correlation matrix.

	Total T	A4	Free T	SHBG
Total T	1			
A4	**0.92** ^∗^	1		
Free T	**0.82** ^∗^	**0.83** ^∗^	1	
SHBG	−0.05	−0.21	**−0.58** ^∗^	1
E2	**0.45** ^∗^	**0.42** ^∗^	0.30	0.11
E1	**0.54** ^∗^	**0.60** ^∗^	**0.47** ^∗^	−0.12
LH	**0.59** ^∗^	**0.57** ^∗^	**0.51** ^∗^	−0.06
FSH	−0.10	−0.14	−0.28	0.35
LH/FSH	**0.61** ^∗^	**0.61** ^∗^	**0.62** ^∗^	−0.21

Data are reported as Spearman's *ρ*. ^∗^ *P* < 0.05 after the Bonferroni correction. Total T: total testosterone; A4: androstenedione; free T: calculated free testosterone; SHBG: sex hormone-binding globulin; E2: estradiol; E1: estrone; LH: luteinizing hormone; FSH: follicle-stimulating hormone.

**Table 3:** Associations between androgens and SHBG and metabolic and ovarian parameters.

	Adjustments	Total T	A4	Free T	SHBG
BMI	Unadjusted	1.06	1.17	**2.09**	**−2.89**
		(−0.13, 2.24)	(−0.001, 2.34)	**(0.98, 3.19)** ^∗∗∗^	**(−4.16, −1.61)** ^∗∗∗^
	Age	1.06	1.17	**2.12**	**−2.92**
		(−0.13, 2.25)	(−0.01, 2.35)	**(1.00, 3.23)** ^∗∗∗^	**(−4.21, −1.64)** ^∗∗∗^

Glucose	Unadjusted	0.07	0.25	0.02	−1.50
		(−1.68, 1.81)	(−1.50, 1.99)	(−0.02, 0.06)	(−3.64, 0.65)
	Age and BMI	−0.19	−0.01	0.01	−0.90
		(−1.95, 1.57)	(−1.78, 1.76)	(−0.03, 0.05)	(−3.40, 1.61)

Insulin	Unadjusted	3.89	4.53	**0.01**	**−19.39**
		(−5.67, 13.45)	(−4.98, 14.04)	**(0.002, 0.02)** ^∗^	**(−30.20, −8.58)** ^∗∗^
	Age and BMI	−0.79	−0.63	0.001	−5.52
		(−8.33, 6.76)	(−8.19, 6.94)	(−0.007, 0.01)	(−16.16, 5.13)

HOMA-IR	Unadjusted	0.08	0.09	**0.48**	**−0.36**
		(−0.10, 0.25)	(−0.09, 0.27)	**(0.10, 0.85)** ^∗^	**(−0.55, −0.16)** ^∗∗^
	Age and BMI	−0.01	0.001	0.11	−0.10
		(−0.14, 0.13)	(−0.14, 0.14)	(−0.38, 0.60)	(−0.29, 0.09)

Total cholesterol	Unadjusted	**9.95**	**7.22**	**0.01**	1.92
		**(3.56, 16.33)** ^∗∗^	**(0.64–13.80)** ^∗^	**(0.001, 0.02)** ^∗^	(−6.35, 10.20)
	Age and BMI	**8.90**	6.00	0.01	8.19
		**(2.41, 15.38)** ^∗∗^	(−0.70, 12.71)	(−0.003, 0.01)	(−0.95, 17.31)

HDL cholesterol	Unadjusted	1.24	0.09	−0.01	**4.65**
		(−2.03, 4.52)	(−3.19, 3.37)	(−0.03, 0.01)	**(0.84, 8.47)** ^∗^
	Age and BMI	1.88	0.80	−0.004	**4.42**
		(−1.27, 5.03)	(−2.39, 3.98)	(−0.02, 0.01)	**(0.22, 8.62)** ^∗^

LDL cholesterol	Unadjusted	**7.17**	**5.96**	**0.01**	0.08
		**(1.66, 12.67)** ^∗^	**(0.39, 11.52)** ^∗^	**(0.001, 0.02)** ^∗^	(−6.93, 7.08)
	Age and BMI	**6.01**	4.67	0.01	5.51
		**(0.45, 11.57)** ^∗^	(−0.98, 10.32)	(−0.004, 0.02)	(−2.23, 13.25)

Triglycerides	Unadjusted	7.69	5.88	**0.01**	**−14.03**
		(−3.78, 19.17)	(−5.62, 17.37)	**(0.001, 0.01)** ^∗^	**(−27.68, −0.39)** ^∗^
	Age and BMI	5.04	2.65	0.005	−8.70
		(−6.04, 16.12)	(−8.50, 13.80)	(−0.0004, 0.01)	(−23.76, 6.35)

AMH	Unadjusted	**0.08**	**0.08**	**0.06**	−0.01
		**(0.03, 0.12)** ^∗∗^	**(0.03, 0.12)** ^∗∗^	**(0.02, 0.11)** ^∗∗^	(−0.05, 0.03)
	Age and BMI	**0.07**	**0.08**	**0.06**	−0.01
		**(0.03, 0.12)** ^∗∗^	**(0.03, 0.12)** ^∗∗^	**(0.02, 0.10)** ^∗∗^	(−0.05, 0.03)

Ovarian volume	Unadjusted	**0.15**	**0.17**	**0.16**	−0.05
		**(0.09, 0.22)** ^∗∗∗^	**(0.11, 0.23)** ^∗∗∗^	**(0.09, 0.22)** ^∗∗∗^	(−0.11, 0.01)
	Age and BMI	**0.15**	**0.17**	**0.14**	−0.03
		**(0.08, 0.21)** ^∗∗∗^	**(0.11, 0.23)** ^∗∗∗^	**(0.08, 0.20)** ^∗∗∗^	(−0.09, 0.02)

AFC	Unadjusted	**0.04**	**0.05**	**0.04**	−0.01
		**(0.03, 0.06)** ^∗∗∗^	**(0.03, 0.06)** ^∗∗∗^	**(0.02, 0.05)** ^∗∗∗^	(−0.03, 0.005)
	Age and BMI	**0.04**	**0.05**	**0.04**	−0.01
		**(0.03, 0.06)** ^∗∗∗^	**(0.03, 0.06)** ^∗∗∗^	**(0.02, 0.05)** ^∗∗∗^	(−0.02, 0.003)

Linear regression with adjustments for age and BMI. Data are reported as *β* coefficients with 95% confidence interval per standard deviation increase in androgen/SHBG. ^∗^ *P* < 0.05, ^∗∗^ *P* < 0.01, ^∗∗∗^ *P* < 0.001. T: testosterone; A4: androstenedione; SHBG: sex hormone-binding globulin; BMI: body mass index; HOMA-IR: homeostasis model assessment of insulin resistance; AMH: anti-Mullerian hormone; AFC: antral follicle count.

**Table 4:** Comparison of normal versus high free T in the study sample.

	Free T ≤ 0.49 ng/dL; *n* = 37 (56%)	Free T > 0.49 ng/dL; *n* = 29 (44%)	*P* value unadjusted	*P* value (age and BMI adjusted)
Clinical parameters				
Age	28.6 (2.4)	28.0 (3.7)	0.419	
Hirsutism score	1.5 (1.9)	2.9 (2.8)	**0.029**	0.262
Having hirsutism (%)	9 (25%)	12 (42.9%)	0.131	0.515
Having acne (%)	16 (46%)	12 (41%)	0.728	0.902
Hormones				
Total T (ng/dL)	33.3 (14.7)	63.3 (22.5)	**<0.001**	**<0.001**
A4 (ng/dL)	127 (52)	247 (83)	**<0.001**	**<0.001**
Free T (ng/dL)	0.32 (0.12)	0.90 (0.33)	**<0.001**	**<0.001**
SHBG (nmol/L)	85.1 (33.0)	50.9 (19.6)	**<0.001**	**0.001**
E2 (ng/L)	56.7 (48.9)	62.3 (53.3)	0.657	0.878
E1 (ng/L)	54.7 (31.5)	71.1 (34.0)	**0.047**	0.131
LH (U/L)	7.9 (3.9)	11.3 (5.3)	**0.005**	**0.008**
LH/FSH	1.3 (0.7)	2.1 (0.9)	**<0.001**	**0.001**
AMH (ng/mL)	6.9 (4.5)	10.7 (5.9)	**0.007**	**0.005**
Metabolic parameters				
BMI	23.3 (4.3)	27.2 (5.5)	**0.003**	
% normal BMI	24 (65%)	12 (41%)		
% overweight	11 (30%)	10 (34%)		
% obese	2 (5%)	7 (24%)		
Glucose (mg/dL)	89.8 (8.0)	91.3 (6.6)	0.451	0.911
Insulin (pmol/L)	55.8 (31.6)	82.6 (46.3)	**0.010**	0.450
HOMA-IR	1.03 (0.57)	1.53 (0.85)	**0.008**	0.408
Ovarian parameters				
Mean ovarian volume (mL)	8.0 (2.8)	12.2 (4.2)	**<0.001**	**<0.001**
Mean AFC	23.9 (10.5)	39.1 (15.0)	**<0.001**	**<0.001**
Follicle localization			**0.047**	
Random	23 (74%)	10 (42%)		
Peripheral	7 (23%)	13 (54%)		
Random + peripheral	1 (3%)	1 (4%)		
Follicle size			**0.007**	
Uniform	6 (21%)	12 (60%)		
Nonuniform	23 (79%)	8 (40%)		
% of women with PCOM	27 (87%)	27 (100%)	0.053	

Data are reported as mean (standard deviation) for continuous variables or as *n* (percentage) for categorical variables. Linear or logistic regression was used to assess differences between groups (unadjusted, adjusted for age, and BMI). Total T: total testosterone; A4: androstenedione; free T: calculated free testosterone; SHBG: sex hormone-binding globulin; E2: estradiol; E1: estrone; LH: luteinizing hormone; FSH: follicle-stimulating hormone; HOMA-IR: homeostasis model assessment of insulin resistance; AFC: antral follicle count; PCOM: polycystic ovarian morphology.

## Supplementary Materials

Supplementary Materials Supplementary Figure 1: flow chart of participants. Supplementary text: detailed description of liquid chromatography-tandem mass spectrometry method for serum total testosterone and androstenedione. Supplementary Table 1: comparison of normal versus high total testosterone.
