# Supplementary material for: Free Testosterone Reflects Metabolic as well as Ovarian Disturbances in Subfertile Oligomenorrheic Women
Source: Int J Endocrinol. 2018 Sep 10;2018:7956951. doi: 10.1155/2018/7956951 (PMC6151847; doi:10.1155/2018/7956951)
Supplement: Supplementary Materials — Supplementary Figure 1: flow chart of participants. Supplementary text: detailed description of liquid chromatography-tandem mass spectrometry method for serum total testosterone and androstenedione. Supplementary Table 1: comparison of normal versus high total testosterone. [file 7956951.f1.docx]

**Supplementary material**

**
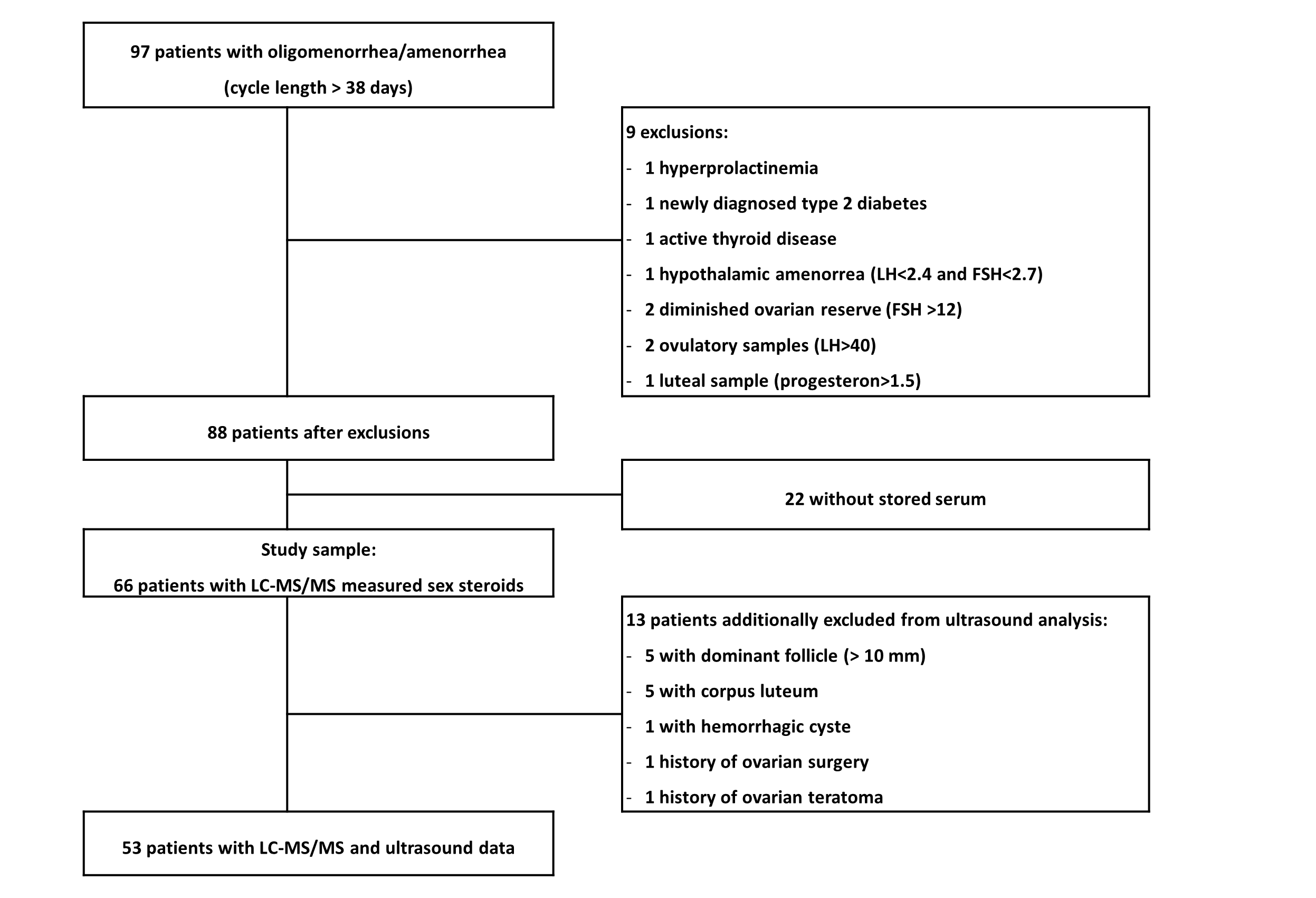
**

**Supplemental Text: Liquid Chromatography tandem Mass Spectrometry Method for Serum Total Testosterone and Androstenedione**

200 µL of serum was precipitated with 400 µL of methanol containing isotopic internal standards (testosterone-2,3,4-^13^C_3_ and androstenedione-2,3,4-^13^C_3_ both at 0.5 ng/mL) in glass tubes. The mixture was vortexed, incubated at room temperature for 1 hour and centrifuged at 2-8°C for 10 min at 1,800x*g*. The supernatant was transferred into a deep well plate and placed into the autosampler. LC-MS/MS analysis was performed on a Sciex QTrap 5500 tandem mass spectrometer combined with a Shimadzu 2 dimensional (2D) chromatography system as described earlier, using the same columns and the same solvents (except that water without fluoride additive was used) [16]. For these parameters, the mass spectrometer was operated in atmospheric pressure chemical ionization (APCI) positive mode at 500°C with nebulizer current set at 3 µA. Multiple reaction monitoring (MRM) was programmed with *m/z* 289.0 🡪109.0 (quantifier) and 289.2🡪97.0 (qualifier) monitored for T; *m/z* 287.1🡪109.0 (quantifier) and 287.0🡪97.0 (qualifier) monitored for A4. For the internal standards, 292.2🡪112.0 (IS1) and 290.1🡪112.0 (IS2) were monitored. Accuracy was assessed by measurement of a reference serum (ERM-DA346a) over three days. The average measured value was 27.47 ng/dL (CV=0.9%), corresponding to +7% difference with the assigned value and well within uncertainty range. The method was linear from 2.5 to 990 ng/dL for total T and from 2.5 to 256 ng/dL for A4. Between-run imprecision (10 measurements, 10 days) was 2.2% at 358.4 ng/dL and 2.9% at 20.8 ng/dL for total T; 3.1% at 54.6 ng/dL and 1.9% at 76.4 ng/dL for A4. LOQ was 2.5 ng/dL for T as well as A4. Carry-over was <0.5% and matrix effect (evaluated by the stability of internal standard peak areas over multiple batches) was found to be very stable between different samples as expected for an APCI method. Published LC-MSMS cutoff values for biochemical hyperandrogenemia were verified by measuring total T and A4 levels (and calculating free T from total T and SHBG by the Vermeulen formula [17]) in serum of 16 young (29-42 years old), normal weight (BMI<25), normal-cycling women, clinically negative for PCOS, that attended the fertility clinic for endometriosis, male subfertility or unexplained infertility. When calculated identically, the cutoffs determined in our verification group were almost identical to published cutoffs for total T (41 ng/dL, identical to published) and free T (0.46 ng/dL, 0.49 ng/dL published). This is in accordance with recent papers describing the good agreement of LC-MSMS total T measurements [9] and supports acceptable agreement of SHBG measurements (used for calculation of free T using identical formula as reference). For A4, the published cutoff deviated somewhat from our verification (203 ng/dL, 240 ng/dL published). This can probably be explained by less stringent standardization of A4 measurements, even for LC-MSMS, as reference sera are not readily available.

**Supplementary table 1: Comparison of normal versus high total testosterone**

|  |  | Normal total T  ≤41 ng/dL  N=30 (45%) | High total T  >41 ng/dl  N=36 (54%) | p-value  unadjusted | p-value  adjusted for age and BMI |
| --- | --- | --- | --- | --- | --- |
|  |  |  |  |  |  |
| **Clinical** | Age | 28.3 (2.6) | 28.2 (3.4) | 0.870 | / |
|  | Hirsutism score | 1.4 (2.3) | 2.6 (2.4) | **0.047** | 0.105 |
|  | Having hirsutism | 16.7% | 44.4% | 0.029 | 0.052 |
|  | Having acne | 30.0% | 52.8% | 0.065 | 0.043 |
|  |  |  |  |  |  |
| **Hormones** | Total T (MS) (ng/dL) | 26.8 (8.1) | 62.9 (19.6) | **<0.001** | **<0.001** |
|  | A4 (MS) (ng/dL) | 109.8 (36) | 239 (78) | **<0.001** | **<0.001** |
|  | E2 (MS) (ng/L) | 54.3 (51.2) | 63.3 (50.3) | 0.474 | 0.552 |
|  | E1 (MS) (ng/L) | 52.0 (33.7) | 70.2 (31.2) | **0.027** | **0.044** |
|  | SHBG (nmol/L) | 73.7 (35.6) | 67.0 (30.0) | 0.411 | 0.785 |
|  | Free T (MS) (ng/dL) | 0.31 (0.14) | 0.79 (0.36) | **<0.001** | **<0.001** |
|  | LH (U/L) | 6.9 (4.3) | 11.5 (4.2) | **<0.001** | **<0.001** |
|  | FSH (U/L) | 6.0 (1.4) | 5.7 (1.1) | 0.307 | 0.381 |
|  | AMH (ng/mL) | 6.1 (4.3) | 10.6 (5.5) | **0.001** | **0.001** |
|  | LH/FSH | 1.1 (0.6) | 2.1 (0.8) | **<0.001** | **<0.001** |
|  |  |  |  |  |  |
| **Metabolic** | BMI | 24.2 (4.6) | 25.7 (5.6) | 0.382 | / |
|  | Glucose (mg/dL) | 91.6 (8.7) | 92.7 (19.5) | 0.285 | 0.200 |
|  | Insulin (pmol/L) | 64.6 (38.1) | 69.6 (42.9) | 0.639 | 0.975 |
|  | HOMA-IR | 1.20 (0.69) | 1.29 (0.79) | 0.632 | 0.981 |
|  |  |  |  |  |  |
| **Ovaries** | Mean ovarian volume (mL) | 7.7 (2.7) | 12.0 (4.1) | **<0.001** | **<0.001** |
|  | Mean AFC | 22.2 (8.9) | 38.3 (14.9) | **<0.001** | **<0.001** |
|  | Follicle localization |  |  | **0.016** |  |
|  | Random | 20 (80%) | 13 (43%) |  |  |
|  | Peripheral | 4 (16%) | 16 (53%) |  |  |
|  | Random+peripheral | 1 (4%) | 1 (3%) |  |  |
|  | Follicle size |  |  | **0.001** |  |
|  | uniform | 3 (12.5%) | 15 (60%) |  |  |
|  | Non-uniform | 21 (87.5%) | 10 (40%) |  |  |
|  | % of women meeting PCOM criteria | 85% | 100% | 0.021 |  |
|  |  |  |  |  |  |

Data are reported as mean (standard deviation) or %. P value: linear regression to compare normal vs elevated testosterone (unadjusted) for continuous variables and logistic regression for categorical variables. Adjusted p-value (age and BMI)

Total T: total testosterone, A4: androstenedione, E2: estradiol, E1: estrone, Free T: calculated free testosterone, IR: insulin resistance, AFC: antral follicle count, PCOM: polycystic ovary morphology
